# Predicting Pregnancy Outcome in Dairy Cows: The Role of IGF-1 and Progesterone

**DOI:** 10.3390/ani13101579

**Published:** 2023-05-09

**Authors:** Nicolae Tiberiu Constantin, Cezar Mihai Bercea-Strugariu, Dragoș Bîrțoiu, Florin Petrișor Posastiuc, Florin Iordache, Liviu Bilteanu, Andreea Iren Serban

**Affiliations:** 1 Department of Clinical Sciences, Faculty of Veterinary Medicine, University of Agronomic Sciences and Veterinary Medicine of Bucharest, 105 Blvd. Splaiul Independentei, 050097 Bucharest, Romania; tiberiu.constantin@fmvb.usamv.ro (N.T.C.); cezarbercea@gmail.com (C.M.B.-S.); dragos.birtoiu@yahoo.ro (D.B.); florin.posastiuc@gmail.com (F.P.P.); 2 Research and Development Institute for Bovine, 077015 Balotesti, Romania; 3 Department of Preclinical Sciences, Faculty of Veterinary Medicine, University of Agronomic Sciences and Veterinary Medicine of Bucharest, 105 Blvd. Splaiul Independentei, 050097 Bucharest, Romania; floriniordache84@yahoo.com; 4 Laboratory of Molecular Nanotechnologies, National Institute for Research and Development in Microtechnologies, 126A Erou Iancu Nicolae, 077190 Voluntari, Romania; 5 Department of Biochemistry and Molecular Biology, Faculty of Biology, University of Bucharest, 91-95 Blvd. Splaiul Independentei, 050095 Bucharest, Romania

**Keywords:** dairy cattle, pregnancy prediction, transition period, IGF-1, progesterone, NEFA, BHB, glucose

## Abstract

**Simple Summary:**

This study aimed to determine the levels of various hormones and metabolites in the blood of Holstein dairy cows during the crucial transition period, 7 days before parturition and a further 14 and 21 days postparturition, exploring the link between pregnancy probability after first artificial insemination and during the first 100 days in milk. The cows were split into two subgroups, those that were diagnosed as pregnant and those that did not conceive after further attempts at artificial insemination. The results showed that the levels of IGF-1 and progesterone exhibited significant differences among the two subgroups, which could have a diagnostic value in predicting pregnancy. These findings are relevant for dairy farm management, as they provide new information about the hormones and metabolites that affect the health and reproductive performance of dairy cows.

**Abstract:**

The purpose of this study was to determine the link between insulin-like growth factor 1 (IGF-1), progesterone (PROG), non-esterified fatty acids (NEFAs), β-hydroxybutyrate (BHB), and glucose (GLU) and pregnancy probability after the first artificial insemination (AI) and during the first 100 days in milk (DIM), during the critical transition period. We determined levels of serum IGF-1, PROG, NEFA, BHB, and GLU in Holstein dairy cows via ELISA, using blood samples collected 7 days before parturition (DAP) until 21 days postparturition (DPP). The group was split into cows diagnosed pregnant at 100 DIM (PREG) and those that did not conceive at 100 and 150 DIM (NPREG). Serum IGF-1 and PROG median levels at 7 DAP were significantly higher in PREG vs. NPREG (*p =* 0.029), the only statistically significant differences across the subgroups. At 7 DAP, IGF-1 levels within the initial group showed a strong negative correlation with PROG (*r =* −0.693; *p =* 0.006), while for the PREG subgroup, the IGF-1 levels exhibited a very strong positive correlation with GLU (*r =* 0.860; *p =* 0.011) and NEFA (*r =* 0.872; *p =* 0.013). IGF-1 and PROG levels detected at 7 DAP may be useful to predict pregnancy at 100 DIM. The positive correlation of NEFA and GLU levels during the transition period demonstrates that the initial group is not in NEB; thus, the NEFA level was not a decisive factor for reproduction success.

## 1. Introduction

Hormonal and metabolic fluctuations during the transition period (±21 days pre- and postcalving) directly affect dairy cows’ health status, productivity, and reproductive performance [1,2,3]. In cows, during the last weeks of gestation, the nutritional needs increase exponentially, along with the fetus and udder enlargement, while the feed intake reduces consequently to the hormonal imbalances that occur due to the onset of parturition. Simultaneously, the vulnerability of this period is closely linked to the sudden increase in energy requirements as a consequence of lactation and reduced dry matter intake (DMI), deepening the negative energy balance (NEB) [2,4]. In cows with increased milk yields, NEB is considered a common disorder during the first 4–6 weeks of lactation. The NEB is characterized by increased energy body reserve mobilization. Proteins and lipids are used to counterbalance the deficit caused by increased milk synthesis.

Along with cholesterol and phospholipids, triglycerides are the main energy sources, being more than 95% of the entire adipose tissue [5]. During periods marked by metabolic stress, such as NEB, free fatty acids or non-esterified fatty acids (NEFAs) are released and mobilized from the body’s fat and transported through albumin linkage through the blood flow to different tissues [6]. The liver has an essential role in lipid metabolism. If the NEB remains, the intake of NEFA overcomes the possibility of complete oxidation in the liver, and the resulting acetyl-CoA is converted into ketone bodies [7] such as acetoacetate, β-hydroxybutyrate (BHB), or acetone, leading to ketosis. NEFAs can also be re-esterified into triglycerides and stored in hepatocytes’ cytoplasm as lipid droplets or exported as very low-density lipoproteins [2,5]. Lipid metabolism imbalances can cause hepatic lipidosis, a subclinical disorder that can affect up to 50% of cows postpartum [8,9]. In addition to ketosis, displacement of the abomasum, which can occur secondary to liver damage, can also disturb fertility [10]. Furthermore, ectopic fat deposited in the deep uterine layer can alter reproductive function. These abnormal deposits can determine low uterine immune response and postpartum uterine subinvolution [8,11]. Moreover, increased levels of NEFA can negatively affect follicular development [12], oocytes, and embryo quality and viability [6,13].

NEFAs could be considered biomarkers for excessive lipid mobilization in high-yielding dairy cows and used to detect early onset of ketosis, displacement of the abomasum, metritis, and mastitis [14]. Alongside NEFA, BHB represents the primary circulating ketone, the leading metabolite used to evaluate dairy cows’ energy profiles [15].

Insulin-like growth factor 1 (IGF-1), produced mainly by the liver [16], mediates puberty [17], postnatal growth, mammary gland development [16], and reproductive functions in cattle. Moreover, IGF-1 is a promoting factor for follicle-stimulating hormone (FSH) and luteinizing hormone (LH) production, essential for proper functioning of the follicles and corpus luteum [18]. In addition, IGF-1 supports steroidogenesis within follicular cells by stimulating the aromatase system, supporting inhibin secretion, and inducing the development of LH receptors on the follicular surface [19]. Although between 18 and 48% of the variations in IGF-1 levels were attributed to genetics, the remaining variation was correlated with the postpartum intake, body weight, calving, parity [16], photoperiodism, and ovarian cycle stage [18]. Postpartum assessment of NEFA and IGF-1 levels has been linked to postpartum disorders [20], while postpartum serum IGF-1 is considered the best predictor for left-displaced abomasum [21]. Thus, during the postpartum period, the uterus is exposed to the animals’ metabolic changes, including decreased glucose (GLU) and IGF-1 levels, followed by diminished neutrophil activity [22] and increased NEFA and BHB levels. Numerous studies [23,24,25] have demonstrated the effects of hormones such as progesterone (PROG), estradiol (E2), and other metabolite parameters of fertility before, during, and after artificial insemination (AI). However, literature regarding the association of the circulating IGF-1 and PROG sequence at 7 days antepartum until 21 days postpartum with fertility in cattle is limited.

Therefore, the overall objective of this study was to determine the association between the levels of IGF-1, PROG, NEFA, BHB, and GLU during the transition period to further examine their effects on the breeding activity onset and performance of cows during the first 150 days in milk. We hypothesized that pregnancy success is associated with significant differences in concentrations of IGF-1, PROG, NEFA, BHB, and GLU at different phases of the transition period, between cow groups, with different reproduction performances evaluated during the first 150 DIM.

## 2. Materials and Methods

### 2.1. Animals and Study Design

The current study started on November 2020 on 17 Holstein dairy cows at the end of gestation, at 7 days antepartum (DAP). Cows were randomly selected from a herd of 1200 heads using free-stall housing (GPS: 43.939° N 24.596° E, Olt County, Romania), being between 1st and 5th lactation.

Feeding consisted of a total mixed ration of 54.1 kg, with 49.1% dry matter (DM), crude protein (CP) of 15.02% DM, acid detergent fiber of 17.90% DM, and non-fiber carbohydrates of 43.09% DM, composed of corn silage (34.80% DM), grain concentrate (42.39% DM), brewer’s grain wheat (9.66% DM), alfalfa hay (4.98% DM), beet molasses (4.49% DM), and alfalfa silage (3.67% DM). This ratio was calculated for 45 L of milk production/cow/day and was administered twice daily, with ad libitum access to water. Cows were milked at 7 a.m. and 5 p.m., with an average milk yield of 37 kg/lactating cow/day at the farm level. The barns were equipped with swinging brushes that were activated following a cow-induced stimulus, while manure was removed using scrapers set to run at 15 min intervals.

The animals were artificially inseminated following estrus synchronization, using semen from EBV bulls that were selected for calving ease, with the pregnancy diagnosis being confirmed after two consecutive transrectal ultrasound checks at 30 and 60 d post-AI. Starting from the 7th month of gestation, the cows received far-off and close-up rations, which were low in concentrates and energy, to allow drying off. With 7 days before the expected calving date, cows were moved to a calving barn, where they were housed on straw bedding. Immediately after calving, calves were allowed to suckle colostrum from their dams, after which they were separated and raised in individual hutches on milk replacements.

Out of the 17 animals that were initially enrolled in the study, three cows had to be excluded from the data analysis because they were culled due to traumatic events that resulted in injuries to their limbs and spinal cord before the end of the experiment. All remaining 14 cows (Table 1) had eutocic calvings, with no retained placenta, and did not manifest any clinical signs suggesting pathological conditions that may affect the results of the study.

After calving, secondary to gynecological examination, the OvSynch [26] protocol was applied starting from 57 days postpartum (DPP), when complete uterine involution and ovarian activity resumption were noted [26]. Subsequently, single straw AI was carried out. At 30 days post-AI, a transrectal ultrasound examination was performed, with a reassessment at 60 days, to exclude embryonic mortality. Cows that did not become pregnant after the first AI were reintroduced to the same protocol until 150 DIM.

### 2.2. Body Condition Score Evaluation

Applying the method described by Edmonson et al. (1989), the body condition score (BCS) was calculated on a scale of 1 to 5, with 0.25-unit increments, yielding a 17-point scoring system. During subject selection, the BCS was determined through visual examination from the back of each cow, with 1 indicating an emaciated animal, while a score of 5 was allocated to an overweight animal [27].

### 2.3. Blood Analysis

The farm’s veterinarian collected blood samples at 7 DAP and 14 and 21 DPP from a coccygeal blood vessel using clot-activator tubes (BD Vacutainer, China) to reduce animal stress. Blood samples were collected after morning milking. After collection, serum tubes were left undisturbed for about 2 h to allow clot formation, centrifuged at 1500× *g* for 15 min at 4 °C, and the serum was harvested and frozen at −80 °C until assayed.

PROG, BHB, and IGF-1 were quantified with bovine ELISA kits produced by Cusabio (Wuhan, China) and GLU and NEFA with bovine ELISA kits from MyBioSource (Eersel, the Netherlands). Spectrometric analysis was performed using a microplate reader (PR 4100 Absorbance Microplate Reader, Magellan software V7.0.5.0, Bio-Rad, Hercules, CA, USA) at 450 nm and with a differential filter at 630 nm. The sensitivity of IGF-1 assay was 0.75 ng/mL with a coefficient of variation (CV) of 3.4 to 5.4%. The PROG assay had a sensitivity of 0.2 ng/mL and the CV ranged from 2.0 to 4.9%. In the case of BHB assay, the sensitivity was less than 9.5 nmol/mL and the CV ranged from 1.7 to 5.9%. Regarding NEFA assay, the sensitivity was 1.2 µmol/L with a CV of 2.7 to 6.9%. Finally, the GLU assay had a sensitivity of 0.055 mmol/L with a CV of 4.7 to 8.8%.

### 2.4. Statistics and Data Analysis

Statistical calculations were performed using the IBM SPSS Statistics 26 software (IBM Corp., Armonk, NY, USA). For all the variables studied, the following descriptive statistics were calculated: mean, 95% confidence interval for the mean, 5% trimmed mean, median, variance, standard deviation, minimum, maximum, range, interquartile range, skewness, and kurtosis. In addition, Kolmogorov–Smirnov and Shapiro–Wilk tests were also applied to determine whether these variables were normally distributed. Descriptive statistics and normality tests were applied to the initial group of *n* = 14 cows (labeled ALL), as well as to the two subgroups: the first subgroup comprised cows that were pregnant (labeled PREG, *n* = 7), while the second subgroup consisted of cows that failed to conceive (labeled NPREG, *n* = 7) by 100 DIM.

Given that the values of the variables were not normally distributed under any circumstances, neither in the case of pregnant cows (PREG) nor for the subgroup of nonpregnant cows (NPREG), non-parametric comparison tests were applied for various purposes: to compare the distributions (Mann–Whitney U test, Kolmogorov–Smirnov test), to compare the means across groups (independent median test), and to estimate the confidence interval of median difference across the groups (Hodges–Lehmann). Finally, the threshold of statistical significance was established at *p =* 0.05.

Since none of the variables were normally distributed, correlation coefficients were calculated using the Spearman scheme in all three groups, ALL, PREG, and NPREG, separately 7 days antepartum (DAP) and 14 and 21 days postpartum (DPP). If the absolute values of these correlation coefficients were in the range 0.75–1.00, they were labeled as “very strong”; if these correlation coefficients were in the range 0.50–0.74, they were labeled as “strong.” The threshold of statistical significance was established at *p =* 0.05.

### 2.5. Ethical Statement

The research activities followed the European Union’s Directive for animal experimentation (Directive 2010/63/EU). The protocol related to the current study (509/01.02.2020) was approved by the Research and Development Institute for Bovine, Balotești, Romania’s Animal Care Committee, and the study was carried out strictly following these procedures.

## 3. Results and Discussions

Successful artificial insemination (AI) was achieved in PREG cows at 100 days in milk (DIM) using a single insemination straw for each animal. Moreover, all cows had maintained their pregnancy at 150 DIM. On the other hand, NPREG cows failed to conceive at both 100 DIM and 150 DIM, with an average of 3.3 ± 0.5 straws used for AI in this subgroup. The median body condition score (BCS) was similar between the two groups (Table 1). In addition, the median milk yield at 100 DIM in the NPREG subgroup, compared to the PREG subgroup, did not display relevant differences, being 3731 and 4241 kg (*p =* 0.109) (Table 1), suggesting that early gestation onset does not influence milk production. These results are in agreement with those published by Roche (2003) [28].

Median serum IGF-1 levels tested at 7 DAP (Table 2) were significantly higher (*p =* 0.029) in cows from the PREG subgroup (48.6 ng/mL), compared to the NPREG subgroup, which registered a median level of 12.0 ng/mL. Overall, the median serum IGF-1 values were significantly higher (*p =* 0.001) antepartum (34.58 ng/mL) than postpartum (2.9 and 3.4 ng/mL at 14 and 21 DPP, respectively) (Table 2). The samples collected at 14 and 21 DPP revealed similar median serum IGF-1 in both groups (*p =* 1.00) (Table 2). In a study performed on lactating Holstein cows, the serum IGF-1 concentration determined at 7 DPP had a median of 31 ng/mL, a value considered to be the optimum serum IGF-1 threshold predictive of pregnancy to first artificial insemination ratio (P/AI) for multiparous cows [16].

When cows were grouped into either high or low IGF-1 categories (greater or less than 31.0 ng/mL), cows with high IGF-1 had 1.61 times greater odds of P/AI than those with low IGF-1. However, pregnancy risk up to 150 and 250 DPP and no significant variations regarding IGF-1 levels in multiparous cows between the considered groups were noted [16]. In our study, the median level of IGF-1 at 14 and 21 DPP (Table 2) was tenfold lower than those previously mentioned. The present results depict a slow increase in IGF-1 at 21 DPP for the PREG cow subgroup compared to 14 DPP (*p =* 0.028). In addition, our data support Falkenberg et al. (2008) [29], who concluded that the circulating IGF-1 at early postpartum stages (1, 4, 10, 20, or 40 DPP) had a minimal diagnostic value in predicting P/AI in multiparous cows.

Our results demonstrated that the IGF-1 levels detected at 7 DAP could have a diagnostic value in predicting pregnancy at 100 DIM, with the differences between groups being statistically significant (*p =* 0.029) (Table 2). Serum IGF-1 concentrations are generally higher in young animals because they are still growing; thus, IGF-1 is strongly correlated to skeletal and muscle development [30]. Gobikrushanth et al. (2018) noted the same patterns by comparing the median levels of IGF-1 from primiparous and multiparous cows (85.0 vs. 31.0 ng/mL) at 7 DPP [16]. In our study, the significant differences recorded in IGF-1 serum concentrations between the two groups at 7 DAP cannot be attributed to parity or age because there are no significant differences regarding both variables (Table 1). Furthermore, Adrien et al. (2012) [31] did not find differences in serum IGF-1 concentrations between primiparous and multiparous cows. Additionally, concerning multiparous cows, a marked decrease in IGF-1 on the day of calving was identified, after which the level remained relatively constant for 30 days. Similarly, our results indicated that the IGF-1 levels’ evaluation is relevant antepartum. Moreover, after calving, a marked decrease in IGF-1 serum concentrations has been proven according to Mihandoost et al. (2019) [18] and Meikle et al. (2004) [32]. The latter have revealed notable IGF-1 level reduction in primiparous cows in comparison with multiparous cows [32].

At 7 DAP, IGF-1 levels within the initial group showed a strong negative correlation with PROG (*r =* −0.693; *p =* 0.006). Moreover, for the PREG subgroup, IGF-1 levels revealed a very strong positive correlation with GLU (*r =* 0.860; *p =* 0.011) and NEFA (*r =* 0.872; *p =* 0.013). Within the NPREG subgroup, the only correlation at 7 DAP was between IGF-1 and BHB (*r =* 0.855, *p =* 0.014) (Table 3).

At 14 DPP, IGF-1 levels do not significantly correlate with PROG. Without considering group separation, a strong positive correlation was established between IGF-1 and GLU levels (*r =* 0.874; *p* < 0.001). Nevertheless, for the NPREG subgroup, a strong positive correlation between IGF-1 and GLU values (*r =* 0.979; *p* < 0.001) has been noted, along with the ones established between IGF-1 and NEFA levels (*r =* 0.814; *p =* 0.026) (Table 3).

At 21 DPP, the correlations between IGF-1 and GLU levels are preserved. However, IGF-1 and NEFA levels’ correlations were not consistent within the NPREG subgroup, while a strong positive one has been established in the PREG subgroup (*r =* 0.979; *p* < 0.001). In addition, at this time interval, a strong negative correlation was observed between IGF-1 and BHB (*r =* −0.811; *p =* 0.027) (Table 3).

Correlation analysis was extended to individual parameter processing at each time interval (7 DAP, 14 DPP, 21 DPP). Thus, the levels of IGF-1 at 7 DAP, for the PREG group, were strongly positively correlated with the levels detected at 21 DPP (*r =* 0.877, *p =* 0.010) (Table 4). This fact is backed up by the slight increase in IGF-1 levels at 21 DPP.

In a recent study, the authors analyzed the levels of IGF-1 and other metabolic parameters weekly, starting from 21 DAP to 21 DPP, and calculated the correlations between these parameters over the entire studied interval [33]. Similarly to our study, IGF-1 was negatively correlated with PROG (*r =* −0.403; *p =* 0.001), whilst it was positively correlated with GLU (*r =* 0.312; *p =* 0.029). The difference between the current study and the above-mentioned study is that our correlations have been considered between the studied parameters for each interval, while in the previously mentioned study, the entire time interval was scrutinized. As is depicted, in our study, the negative correlation between IGF-1 and PROG was statistically significant only for the 7 DAP interval and, for the rest of the analyzed intervals, no statistical relevance has been detected. However, the positive correlation between IGF-1 and GLU remained statistically significant for the NPREG subgroup at 14 and 21 DPP and only at 7 DAP for the PREG subgroup (Table 3).

In order to characterize the transition period in cows, the hormonal and metabolic profile must be discussed for at least two time intervals, antepartum and postpartum. Mikula et al. (2021) also found that IGF-1 was moderately positively correlated with both NEFA (*r =* 0.495; *p =* 0.001) and BHB (*r =* 0.368, *p =* 0.009), positive correlations that our study also found, particularly for the PREG subgroup (Table 3) [33].

Regarding progesterone dynamics before and after calving (Table 2), the median serum concentration at the first sampling, within both subgroups, was statistically significant at 7 DAP (13.4 ng/mL) in comparison with those detected at 14 DPP (2.0 ng/mL, *p =* 0.001) and 21 DDP (2.1 ng/mL, *p =* 0.001). Interestingly, the PREG subgroup has registered lower median levels than the NPREG subgroup (10.0 vs. 16.1 ng/mL, *p =* 0.029) (Table 2). Thus, our study reports for the first time that the levels of this hormone and IGF-1 measured at 7 DAP can be considered promising biomarkers for postcalving fertility in dairy cows.

At 14 and 21 DPP, the PROG median levels of the NPREG subgroup decreased significantly compared to 7 DAP to 1.99 and 2.08 ng/mL, respectively (*p =* 0.018) (Table 2). A similar decrease in PROG level compared to the 7 DAP interval was also recorded in the case of the PREG subgroup, although the detected PROG levels were slightly higher (2.5 and 2.3 ng/mL, *p =* 0.018) (Table 2). PROG is generally used as a marker of fertility (P/AI) or for estrus detection, evaluated before, during, and after AI [24,25,34].

In a recent study that evaluated hormonal changes during the transition period in cows, PROG levels registered a V-shaped curve during the experimental trial, with the highest levels being at 21 DAP [33]. On the other hand, at 7 DAP, the detected PROG value was approximately 3.3 times lower than those reported in the present paper. In addition, at 14 DPP, the PROG levels had similar values in both studies; at 21 DPP, the presented results showed that PROG registered a 1.5-fold decrease [33]. However, Mikula et al. (2021) did not report the correlation of the PROG levels with AI success at 100 or 150 DIM [33]. Although, at 14 and 21 DPP, the mean levels of PROG did not differ significantly between the two subgroups (NPREG vs. PREG), PROG concentrations were slightly higher in the PREG subgroup (Table 2). Moreover, we did not find any statistically significant pairwise correlation between the 7 DAP, 14 DPP, and 21 DPP PROG levels within any group of cows (Table 4).

Madureira et al. (2021) showed that cows with a greater intensity of estrus expression at 4 days before AI had greater concentrations of PROG than cows with lower intensity estrus or no estrus expression (4.6 ± 0.2 vs. 3.6 ± 0.2 vs. 3.7 ± 0.2 ng/mL). They concluded that higher concentrations of PROG before and during AI were associated with greater estrus intensity and P/AI at spontaneous and timed AI events [23]. As has been stated above, at 7 DAP, the PROG levels showed a strong negative correlation with IGF-1 (*r =* −0.693; *p =* 0.006); otherwise, no other significant correlation was detected among the analyzed metabolites at the studied intervals (Table 3). However, Mikula et al. (2021) showed that PROG was moderately negatively correlated with both NEFA (*r =* 0.45; *p =* 0.002) and BHB (*r =* 0.34, *p =* 0.018) in a time interval between 21 DAP and 21 DPP [33].

The NEFA, BHB, and GLU levels evaluated in the current study did not show significant differences (*p* > 0.05) between the two subgroups in any of the three sampling moments (Table 2). However, the only statistically significant increase was recorded within the initial group for the BHB levels between 7 DAP and 14 DPP (*p =* 0.035), as well as for NEFA levels at 14 DPP compared to those at 21 DPP (*p =* 0.026) (Table 2). Nevertheless, the BHB and NEFA levels were lower than those reported in other studies, especially during the postpartum period [31,35], when NEB occurred. In the previously mentioned studies, the increase in NEFA and BHB was correlated with a slight decrease in GLU levels during the first postpartum weeks.

Regarding the effects of the reduced postpartum dietary energy levels starting with the day 0 dry period on resumption of ovarian cyclicity and reproductive performance, similar postpartum NEFA and GLU levels were reported [36]. Thus, the current findings might result from a balanced nutrition plan and the good health status of the animals in our study. Another hypothesis regarding the lack of differences within and between the two subgroups could be explained by the fact that the trial was performed in one month (November). Thus, this approach may elude variations due to climatic, managerial, or nutritional changes that might occur during different seasons under temperate climates and neutral thermo-climatic conditions for lactating dairy cows [16].

Cows with many days open (>130 d), or cows that did not get pregnant at all, had greater plasma concentrations of NEFA as well as a more severe NEB than cows with few (<80 d) days open [36]. Moreover, it was concluded that a low plasma NEFA level, along with a positive EB, may have been related to an earlier postovulatory PROG increase, a greater follicle development, and a better resumption of ovarian activity, resulting in fewer days open [36]. In addition, it has been stated that high NEFA levels during the transition period were associated with a decreased pregnancy rate at first AI [12] or at 70 d after the voluntary waiting period [4]. Another study concluded that high circulating NEFA was associated with a reduced 21 d pregnancy rate after a voluntary waiting period [4]. High NEFA values during negative EB may prevent follicle development, interrupt the endocrine system, and promote ovarian cysts [18]. These results did not agree with our study: NEFA levels were low throughout the studied interval and did not differ statistically between the PREG and NPREG subgroups, at any analyzed time interval, thus NEFA levels have not been significant for reproduction success.

Within the initial group, as well as for the PREG subgroup, GLU levels are strongly positively correlated with the NEFA levels, at all of the studied intervals (Table 3). For the PREG subgroup, the dynamics of NEFA levels exhibited strong positive correlations between different time intervals (Table 4). Similar results have been obtained for GLU levels (Table 4). The fact that NEFA and GLU levels at all time intervals were positively correlated proves that both the initial group and the PREG subgroup were not in NEB, otherwise NEFA and GLU levels would have been negatively correlated. A recent study, that investigated the changes in metabolic and hormonal profiling during the transition period in dairy cattle, concluded that the NEFA levels were moderately positively correlated with BHB levels (*r =* 0.430 and *p =* 0.002), and BHB levels were moderately negatively correlated with GLU levels (*r =* −0.397 and *p =* 0.004), highlighting the development of NEB [33].

Overall, these findings contribute to our understanding of the physiological changes that occur during the transition period in dairy cattle and have the potential to inform management strategies that could improve reproductive performance and overall productivity in this important sector. Further research is needed to validate these findings and explore the potential of these markers as tools for reproductive management in dairy cattle.

## 4. Conclusions

In conclusion, the significant differences in serum IGF-1 and PROG levels between the pregnant and non-pregnant subgroups at 7 days before parturition highlight the potential of these assays as diagnostic markers for pregnancy at 100 days in milk (DIM). The positive correlation between NEFA and GLU levels during the transition period suggests that the initial group is not in negative energy balance, making NEFA level not a determining factor for reproductive success.

## Figures and Tables

**Table 1:** Descriptive statistics of some clinical variables for the group including all dairy cows and for the two experimental subgroups PREG and NPREG.

	All	Non-Pregnant		Pregnant
Effective, N	14	7		7
Age (in months)				
Median		44.88	48.48	39.48
			*p =* 0.593	
Minimum		9.12	33.60	9.12
Maximum		75.24	75.24	69.12
Parity				
Median		3.00	3.00	2.00
			*p =* 0.515	
Minimum		1.00	2	1.00
Maximum		5.00	5	4.00
Milk yield (L) in the first 100 DIM				
Median		3796	3731	4241
			*p =* 0.109	
Minimum		3250	3250	3410
Maximum		5360	4970	5360
BCS				
Median		3.25	3.25	3.25
			*p =* 0.611	
Minimum		3.25	3.25	3.25
Maximum		3.50	3.50	3.50

*p*—asymptotic significance (2-sided test); BCS—body condition score, DIM—days in milk, NPREG—non-pregnant cows group, PREG—pregnant cows group.

**Table 2:** Descriptive statistics of the levels of hormones and metabolites measured at the different times (7 DAP, 14 DPP, and 21 DPP) of the transition period for ALL dairy cows included and for the two experimental groups PREG and NPREG. Various tests were performed to compare the median levels of these metabolites between the NPREG and PREG groups, and within the ALL, NPREG, and PREG groups, comparisons were made between the levels obtained at 7 DAP and 14 DPP, at 7 DAP and 14 DPP, and at 14 DPP and 21 DPP, respectively.

	7 DAP			14 DPP			21 DPP		
	ALL	NPREG	PREG	ALL	NPREG	PREG	ALL	NPREG	PREG
Insulin-like growth factor 1 (ng/mL)									
Median	34.6	12.0	43.6	2.9	3.1	2.6	3.3	3.3	3.3
IQR	32.9	13.1	16.9	1.2	1.0	1.8	1.1	0.7	1.8
*p* ^(1)^		0.029			1.000			1.000	
*p* ^(2)^				0.001	0.028	0.018	0.001	0.018	0.018
*p* ^(3)^							0.048	0.499	0.028
Progesterone (ng/mL)									
Median	13.4	16.1	10.0	2.1	2.0	2.5	2.1	2.1	2.3
IQR	6.9	10.1	4.9	1.0	0.3	1.7	0.8	0.6	1.2
*p* ^(1)^		0.029			0.286			1.000	
*p* ^(2)^				0.001	0.018	0.018	0.001	0.018	0.018
*p* ^(3)^							0.826	0.310	0.612
β-hydroxybutyrate (nmol/mL)									
Median	67.1	65.3	68.9	72.9	75.1	71.7	71.9	73.5	71.3
IQR	28.3	31.5	22.5	14.9	31.1	14.1	9.5	14.8	3.6
*p* ^(1)^		1.000			1.000			1.000	
*p* ^(2)^				0.035	0.237	0.091	0.673	0.735	0.499
*p* ^(3)^							0.551	0.683	0.176
Glucose (mmol/L)									
Median	2.1	2.5	1.6	1.9	1.9	2.0	2.4	1.9	2.4
IQR	3.7	3.7	4.8	2.3	0.8	4.3	4.1	2.6	3.5
*p* ^(1)^		0.286			1.000			1.000	
*p* ^(2)^				0.638	0.237	0.499	0.975	0.812	0.499
*p* ^(3)^							0.470	0.866	0.091
Non-esterified fatty acids (µmol/L)									
Median	114.3	133.3	112.3	100.0	92.4	105.4	112.9	94.2	122.7
IQR	97.5	106.0	97.8	57.2	60.8	77.2	65.5	83.8	50.5
*p* ^(1)^		1.000			0.286			1.000	
*p* ^(2)^				0.064	0.866	0.310	0.470	0.866	0.398
*p* ^(3)^							0.026	0.735	0.176

ALL—the group containing all the cows, DAP—days before parturition, DPP—days postparturition, IQR—interquartile range, NPREG—non-pregnant cows group, PREG—pregnant cows group; *p* ^(1)^—Fisher exact significance (2-sided test) of independent samples median test applied to the distribution of values from the PREG and NPREG subgroups; *p* ^(2)^—statistical significance of the Wilcoxon signed rank test comparing the distributions of the values corresponding to the initial group (ALL) and the PREG and NPREG subgroups measured at 7 DAP vs. 14 DPP and 7 DAP vs. 21 DPP, respectively; *p* ^(3)^—statistical significance of the Wilcoxon signed rank test comparing the distributions of values corresponding to the initial group (ALL) and the PREG and NPREG subgroups measured at 14 DPP vs. 21 DPP.

**Table 3:** Spearman correlation coefficients (*r*) between the levels of different hormones and metabolites measured at 7 DAP, 14 DPP, and 21 DPP for all 3 groups: ALL, PREG, and NPREG. The values of the correlation coefficients are color-coded: green corresponds to high positive values (the higher the value, the darker the green), yellow corresponds to moderately positive values; red corresponds to negative values (the lower the value, the darker the red).

ALL				PREG				NPREG					
		*r*	*p*			*r*	*p*				*r*	*p*	
7 DPP													
IGF-1	Progesterone	−0.693	0.006	IGF-1	Glucose	0.860	0.013	IGF-1		BHB	0.855	0.014	
Glucose	NEFA		0.888		<0.001	IGF-1		NEFA		0.872	0.011		
						Glucose		NEFA		0.985	<0.001		
14 DAP													
IGF-1	Glucose	0.874		<0.001	Glucose	NEFA	0.982	<0.001	IGF1	Glucose		0.979	<0.001
Glucose		NEFA		0.820	<0.001					IGF1	NEFA	0.814	0.026
21 DAP													
IGF-1	Glucose	0.749	0.002	IGF-1	NEFA		0.979	<0.001	IGF-1	BHB	−0.811	0.027	
IGF-1	NEFA	0.810	<0.001	Glucose	NEFA		0.750	0.050	IGF-1	Glucose	0.778	0.040	
Glucose	NEFA	0.775	<0.001						Glucose	NEFA	0.857	0.014	

*p*—Fisher exact significance (2-sided test); ALL—the group including all the cows; BHB—β-hydroxybutyrate; IGF-1—insulin-like growth factor 1; DAP—days antepartum; DPP—days postpartum; NEFA—non-esterified fatty acid; NPREG—the subgroup of non-pregnant cows; PREG—the subgroup of pregnant cows.

**Table 4:** Spearman correlation coefficients (*r)* between the levels of the same hormone or metabolite measured at different times, 7 DAP, 14 DPP, and 21 DPP, of the transition period for all 3 groups (ALL, PREG, and NPREG). The values of the correlation coefficients are color-coded: green corresponds to high positive values (the higher the value, the darker the green) and yellow corresponds to moderately positive values.

ALL				PREG				NPREG				
		*r*	*p*			*r*	*p*			*r*	*p*	
IGF-1												
7 DAP	21 DPP	0.557	0.038	7 DAP	21 DPP	0.877	0.010					
BHB												
7 DAP	14 DPP	0.817	<0.001	7 DAP	14 DPP	0.784	0.037	7 DAP	14 DPP	0.866	0.012	
Glucose												
7 DAP	21 DPP	0.642	0.013	7 DAP	14 DPP	0.978	<0.001	14 DPP		21 DPP	0.865	0.012
14 DPP	21 DPP	0.728	0.003	7 DAP	21 DPP	0.916	0.004					
				14 DPP	21 DPP	0.860	0.013					
NEFA												
14 DPP	21 DPP	0.889	<0.001	14 DPP	21 DPP	0.981	<0.001	14 DPP		21 DPP	0.793	0.033

*p*—Fisher exact significance (2-sided test); ALL—the group including all the cows; BHB—β-hydroxybutyrate; IGF-1—insulin-like growth factor 1; DAP—days antepartum; DPP—days postpartum; NEFA—non-esterified fatty acid; NPREG—the subgroup of non-pregnant cows; PREG—the subgroup of pregnant cows.

## Data Availability

The datasets used and analyzed during the current study are available from the corresponding author on reasonable request.
